# Supplementary material for: Detection of Zika and dengue viruses in wild-caught mosquitoes collected during field surveillance in an environmental protection area in São Paulo, Brazil
Source: PLoS One. 2020 Oct 16;15(10):e0227239. doi: 10.1371/journal.pone.0227239 (PMC7567345; doi:10.1371/journal.pone.0227239)
Supplement: S1 Table — Sequences of dengue and Zika viruses from GenBank aligned to construct a phylogenetic tree by country of origin, isolate, year, origin of isolated material, genome sequence and GenBank accession number. (DOCX) [file pone.0227239.s001.docx]

**S1 Table. Reference sequences of dengue and Zika viruses from GenBank.**

| **Viral isolation** | | | | **Genome sequence** | |  |
| --- | --- | --- | --- | --- | --- | --- |
| **Country of origin** | **Isolate** | **Year** | **Origin of isolated material** | **Complete** | **Partial** | **GenBank accession number** |
| Thailand | DENV-1 | 2001 | *Homo sapiens* | x |  | FJ687432 |
| Puerto Rico | DENV-1 | 2010 | *Homo sapiens* | x |  | KJ189367 |
| Cuba | DENV-2 | 1891 | *Homo sapiens* | x |  | KF704357 |
| Papua New Guineas | DENV-2 | 1994 | *Homo sapiens* | x |  | KM204118 |
| Brazil | DENV-2 | 2000 | *Homo sapiens* | x |  | JN819419 |
| China | DENV-2 | 2015 | *Homo sapiens* | x |  | KU094070 |
| Colombia | DENV-3 | 2004 | *Homo sapiens* | x |  | GU131953 |
| Brazil | DENV-3 | 2007 | *Homo sapiens* | x |  | GU131878 |
| Cambodia | DENV-3 | 2008 | *Homo sapiens* | x |  | GU131905 |
| Cambodia | DENV-4 | 2002 | *Homo sapiens* | x |  | KF955510 |
| Brazil | DENV-4 | 2012 | *Homo sapiens* | x |  | KP188560 |
| Uganda | ZIKV | 1947 | Sentinel Monkey | x |  | NC012532 |
| Nigeria | ZIKV | 1968 | *Homo sapiens* | x |  | KU963574 |
| Senegal | ZIKV | 1984 | *Aedes taylori* | x |  | MF510857 |
| Micronesia | ZIKV | 2007 | *Homo sapiens* | x |  | EU545988 |
| Filipinas | ZIKV | 2012 | *Homo sapiens* | x |  | KU681082 |
| Brazil | ZIKV | 2015 | *Homo sapiens* | x |  | KU729218 |
| Brazil | ZIKV | 2015 | *Homo sapiens* | x |  | KU497555 |
| United States | ZIKV | 2016 | *Aedes aegypti* | x |  | KY785468 |
| Brazil | ZIKV | 2016 | *Homo sapiens* | x |  | KY014307 |
| Colombia | ZIKV | 2016 | *Homo sapiens* | x |  | MK049249 |
| Venezuela | ZIKV | 2016 | *Homo sapiens* | x |  | KX893855 |
| Haiti | ZIKV | 2016 | *Homo sapiens* | x |  | MF783073 |
| Ecuador | ZIKV | 2016 | *Homo sapiens* | x |  | MF794971 |
| Colombia | ZIKV | 2016 | *Aedes aegypti* | x |  | MK049247 |
| Brazil | ZIKV | 2017 | *Callithrix* sp. |  | x | MG770183 |
| Cuba | ZIKV | 2017 | *Homo sapiens* | x |  | MH063264 |

Sequences of dengue and Zika viruses from GenBank aligned to construct a phylogenetic tree by country of origin, isolate, year, origin of isolated material, genome sequence and GenBank accession number.
