# Supplementary material for: Detection of Zika and dengue viruses in wild-caught mosquitoes collected during field surveillance in an environmental protection area in São Paulo, Brazil
Source: PLoS One. 2020 Oct 16;15(10):e0227239. doi: 10.1371/journal.pone.0227239 (PMC7567345; doi:10.1371/journal.pone.0227239)
Supplement: S2 Table — The table shows the number of individuals at each study point, forest stratum, sex and number of pools. Mosquitoes collected from March 2016 to April 2017. (DOCX) [file pone.0227239.s002.docx]

**S2 Table. Mosquito species collected in the Capivari-Monos Environmental Protection Area (EPA).**

| **Taxa** | **Capivari-Monos EPA** | | | | | | | | | | | |  | | **Sex** | **Total** | | **Pools** | |
| --- | --- | --- | --- | --- | --- | --- | --- | --- | --- | --- | --- | --- | --- | --- | --- | --- | --- | --- | --- |
|  | **Marsilac village** | | **Wild area** | | **Embura village** | | | **Transition area** | | | **No information** | | | |  |  |  |  |  |
|  | **Canopy** | **Ground** | **Canopy** | **Ground** | | **Canopy** | **Ground** | | **Canopy** | **Ground** | | **Canopy** | | **Ground** |  | |  | |  |
| *Aedeomyia ( Aedeomyia) squamipennis* |  |  |  | 1 | |  |  | |  |  | |  | |  | f | | 1 | | 1 |
| *Aedes (Georgecraigius) fluviatilis* |  | 1 | 1 |  | |  |  | |  |  | |  | |  | f | | 2 | | 2 |
| *Aedes (Ochlerotatus) crinifer* |  |  | 2 |  | |  |  | |  | 3 | |  | |  | f | | 5 | | 2 |
| *Aedes (Ochlerotatus) scapularis* |  |  | 1 |  | |  |  | |  |  | |  | | 1 | f | | 2 | | 2 |
| *Aedes (Ochlerotatus) serratus* |  |  | 5 | 10 | |  | 5 | | 1 |  | |  | |  | f | | 21 | | 11 |
| *Aedes (Protomacleaya) terrens* |  |  | 1 |  | |  |  | |  |  | |  | |  | f | | 1 | | 1 |
| *Aedes (Stegomyia) albopictus* |  | 1 |  |  | |  |  | |  |  | |  | |  | f | | 1 | | 1 |
| *Anopheles (Kerteszia) cruzii* | 10 | 1 | 119 | 36 | |  |  | | 3 | 2 | |  | |  | f | | 171 | | 31 |
| *Coquillettidia (Rhynchotaenia) albicosta* |  |  |  |  | |  |  | | 1 |  | |  | |  | f | | 1 | | 1 |
| *Coquillettidia (Rhynchotaenia) albifera* |  |  |  |  | | 1 | 1 | |  |  | |  | |  | f | | 2 | | 2 |
| *Coquillettidia (Rhynchotaenia) chrysonotum/albifera* |  | 1 | 1 |  | |  |  | |  |  | |  | |  | f/m | | 2 | | 2 |
| *Coquillettidia (Rhynchotaenia) venezuelensis* |  | 1 |  |  | |  |  | |  | 2 | |  | | 2 | f | | 5 | | 3 |
| *Culex (Carrollia) iridescens* |  |  | 1 |  | | 1 |  | |  |  | |  | |  | f | | 2 | | 9 |
| *Culex (Culex) chidesteri* |  | 5 | 14 | 4 | | 12 |  | | 2 |  | |  | |  | f | | 37 | | 2 |
| *Culex (Culex) dolosus/eduardoi* |  |  | 5 |  | |  |  | |  | 2 | |  | |  | f | | 7 | | 2 |
| *Culex (Culex) nigripalpus* | 3 |  | 5 | 4 | |  |  | | 2 | 2 | |  | |  | f | | 16 | | 11 |
| *Culex (Culex) quinquefasciatus* |  | 1 | 20 | 9 | |  |  | | 1 |  | |  | |  | f | | 31 | | 10 |
| *Culex (Culex)* spp*.* | 4 | 2 | 41 | 21 | | 28 | 4 | |  | 9 | |  | |  | f | | 109 | | 29 |
| *Culex (Melanoconion) delpontei* |  |  | 1 |  | |  |  | |  |  | |  | |  | f | | 1 | | 1 |
| *Culex (Melanoconion) ribeirensis* | 1 |  | 1 |  | | 1 | 5 | |  |  | |  | |  | f/m | | 8 | | 5 |
| *Culex (Melanoconion)* spp. |  |  | 2 | 3 | |  |  | |  |  | |  | |  | f | | 5 | | 2 |
| *Culex (Melanoconion) vaxus* | 4 |  | 24 | 8 | |  |  | |  |  | |  | |  | f | | 36 | | 8 |
| *Culex (Coronator* group) |  | 1 |  |  | |  |  | |  |  | |  | |  | f | | 1 | | 1 |
| *Limatus durhamii* | 1 | 2 | 9 | 8 | |  | 2 | | 3 | 36 | |  | |  | f | | 61 | | 15 |
| *Mansonia (Mansonia) indubitans* | 1 | 1 |  |  | | 2 |  | | 7 |  | |  | |  | f | | 11 | | 6 |
| *Mansonia (Mansonia) titillans* |  |  |  |  | |  |  | |  | 1 | |  | |  | f | | 1 | | 1 |
| *Runchomyia (Runchomyia) reversa* | 3 | 1 | 6 | 29 | | 6 | 1 | | 2 | 7 | |  | |  | f | | 55 | | 19 |
| *Sabethes (Sabethes) purpureus* |  |  | 1 |  | |  |  | |  |  | |  | |  | f | | 1 | | 1 |
| *Trichoprosopon pallidiventer* | 8 |  |  | 9 | |  | 2 | |  |  | |  | | 1 | f | | 20 | | 9 |
| *Wyeomyia (Phoniomyia) davisi* | 2 |  | 10 | 4 | | 2 | 2 | |  |  | |  | | 4 | f | | 24 | | 9 |
| *Wyeomyia (Phoniomyia) edwardsi* |  |  |  |  | | 2 | 3 | |  |  | |  | |  | f | | 5 | | 2 |
| *Wyeomyia (Phoniomyia) incaudata* |  |  | 7 | 2 | | 1 |  | |  |  | |  | |  | f | | 10 | | 4 |
| *Wyeomyia (Phoniomyia) pallidoventer* | 1 |  | 4 |  | |  |  | |  |  | |  | |  | f | | 5 | | 3 |
| *Wyeomyia (Phoniomyia) palmata* |  |  |  |  | |  |  | | 2 |  | |  | |  | f | | 2 | | 1 |
| *Wyeomyia (Phoniomyia) pilicauda* | 3 |  | 4 |  | | 1 |  | | 2 |  | |  | |  | f | | 10 | | 5 |
| *Wyeomyia (Phoniomyia)* spp*.* | 1 |  |  | 6 | |  |  | |  | 4 | |  | |  | f | | 11 | | 3 |
| *Wyeomyia (Phoniomyia) theobaldi* | 5 | 1 | 20 | 6 | | 13 | 2 | | 5 | 1 | |  | | 5 | f | | 58 | | 16 |
| *Wyeomyia (Prosopolepis) confusa* |  |  | 27 | 54 | | 1 | 17 | | 8 | 23 | |  | | 4 | f | | 134 | | 25 |
| *Wyeomyia ( Spilonympha) mystis* |  |  |  | 1 | |  |  | |  |  | |  | |  | f | | 1 | | 1 |
| *Wyeomyia roucouyana/chalcocephala* |  |  |  |  | |  | 2 | |  |  | |  | |  | f | | 2 | | 1 |
| **Total** | **47** | **19** | **332** | **215** | | **71** | **46** | | **39** | **92** | |  | | **17** |  | | **878** | | **260** |
| **N** | **66** | | **547** | | **117** | | | **131** | | | **17** | | | |  |  | |  | |

Mosquito species collected in the Capivari-Monos EPA according to the number of individuals at each study point, stratification, sex and number of pools. Mosquitoes collected from March 2016 to April 2017.
