# Supplementary material for: Detection of Zika and dengue viruses in wild-caught mosquitoes collected during field surveillance in an environmental protection area in São Paulo, Brazil
Source: PLoS One. 2020 Oct 16;15(10):e0227239. doi: 10.1371/journal.pone.0227239 (PMC7567345; doi:10.1371/journal.pone.0227239)
Supplement: Attachment — Submitted filename: Comments on PONE-D-19-34539.pdf [file pone.0227239.s003.pdf]

44 – “Mosquitoes are competent vectors for viruses…”

46 – no hyphens between virus names

67 – “a priority” not “priorities”

127 – The Gubler et al reference contains many different types of virus detection and therefore the exact methods you used are not clear from that reference. Please describe the methods of your IFA assay.

127 – Is this a focus-forming assay? Is it possible to quantify the number of foci and determine how many infectious virus particles were in the mosquito pool? Were positive controls used to compare to positive results? How was “positive” determined for the IFA? What about negative controls for antibody background staining? Please clarify these points, it will improve confidence in the findings of this study.

131 – What quantification method did you use to determine the amount of viral RNA in your qRT-PCR assay? What was the basis of your standard curve?

137 – are these primers the Flavi S and Flavi AS2 primers used for sequencing (Line 140)? If not please clarify which primers were used and provide sequences

162 – is this also how many were positive by the IFA? Meaning, all the IFA positives were also positive by qRT-PCR?

162 – Since this is a qRT-PCR assay, please report the quantities of viral RNA found in the supernatants of the C636 cells. This will help determine if the virus from the inoculated mosquito pool was infectious and replicated in the C636 cells.

162 – The qRT-PCR protocol you used, as described by Patel et al, is very sensitive (10-100 genome copies). Why not determine the presence of viral RNA directly in the mosquito pools themselves?

206 – Technically this study doesn’t demonstrate that the mosquitoes are infected with the viruses, but that the mosquitoes contain infectious virions (if the IFA is indeed a focus forming assay). Just because there is no visible blood does not mean these mosquitoes have not blood-fed recently and that some infectious virus could remain even if it isn’t infecting the mosquitoes themselves. Clarify language here and in other areas where “infected” is used

258 – Guedes et al do not demonstrate ZIKV replication in *Cx. quinquefasciatus* – a closer look at the titers of virus RNA in those mosquitoes will show RNA levels for ZIKV remaining stagnant over time and do not increase at any point measured. Clarify language

262 – Your study has absolutely not demonstrated this. You have found DENV/ZIKV RNA in cell culture samples that are positive for Flaviviruses by IFA. This indicates that pools of these mosquitoes may contain virions that can infect C636 cells, but does not indicate that these virions are infecting the mosquitoes in the pool, or that they would be disseminated in the mosquito saliva to new hosts. This sentence greatly misrepresents your findings and should be rewritten.

281 – Once again, you have not found these mosquitoes to be “infected”. Clarify language

293 – I’m not sure one citation constitutes this mosquito being found “frequently… in natural and artificial breeding sites”. Possibly clarify this sentence or find more references

295 – The sentence “This mosquito bites actively…” should have citations
