# Supplementary material for: Detection of Zika and dengue viruses in wild-caught mosquitoes collected during field surveillance in an environmental protection area in São Paulo, Brazil
Source: PLoS One. 2020 Oct 16;15(10):e0227239. doi: 10.1371/journal.pone.0227239 (PMC7567345; doi:10.1371/journal.pone.0227239)
Supplement: Attachment — Submitted filename: ReplytoReviewers_rev.docx [file pone.0227239.s004.docx]

To the Editor

Plos One

Dear Dr. Dimopoulos

Thank you for reviewing our manuscript. We have made substantial changes to the scientific note to address the points made by the reviewers. We believe the changes proposed by the reviewers have improved the article significantly.

Sincerely,

Mauro Marrelli

**Answers: Reviewer #1**

We are grateful to the reviewer for his/her very useful comments. All the suggestions and criticisms have been addressed.

**Query 1**

Line 44 – “Mosquitoes are competent vectors for viruses…”

**Query 2**

Line 46 – no hyphens between virus names

**Query 3**

Line 67 – “a priority” not “priorities”

**Answer:** We thank the reviewer for pointing out these mistakes and have made the necessary corrections to the manuscript.

**Query 4**

Line 127 – The Gubler et al reference contains many different types of virus detection and therefore the exact methods you used are not clear from that reference. Please describe the methods of your IFA assay.

**Answer:** A description of the methods used in the IFA has been added.

**Query 5**

Line 127 – Is this a focus-forming assay? Is it possible to quantify the number of foci and determine how many infectious virus particles were in the mosquito pool? Were positive controls used to compare to positive results? How was “positive” determined for the IFA? What about negative controls for antibody background staining? Please clarify these points, it will improve confidence in the findings of this study.

**Answer:** The IFA is not a focus-forming assay. After inoculation of the C6/36 cells with the mosquito pools, the virus replicated (this is why we kept it for 9 days, which is the mean time for dengue virus replication in Vero cell lines). In the IFA the cytoplasm of the cells was fluorescent, showing that the virus was able to replicate in this cell line and was viable, confirming viral isolation. We used the 17DD Yellow Fever virus as positive control, and a non-inoculated tube as negative control.

**Query 6**

Line 131 – What quantification method did you use to determine the amount of viral RNA in your qRT-PCR assay? What was the basis of your standard curve?

**Answer:** We did not quantify the amount of RNA. We followed the approach described in the original article by Patel et al. (2013), who determined that positive results are those with Ct values lower than 39.7. This information has been added in the text.

**Query 7**

Line 137 – are these primers the Flavi S and Flavi AS2 primers used for sequencing (Line 140)? If not please clarify which primers were used and provide sequences

**Answer:** Yes, we used the same primers, as stated in lines 151-152.

"The qRT-PCR amplicons (~260bp) were directly sequenced with the BigDyeTM kit v3.1 (Applied Biosystems, Inc., Foster City, CA, USA) and Flavi S and Flavi AS2 primers [[**26**](https://www.biorxiv.org/content/10.1101/2019.12.17.879643v1.full#ref-26)].

**Query 8**

Line 162 – is this also how many were positive by the IFA? Meaning, all the IFA positives were also positive by qRT-PCR?

**Answer:** Yes. All isolates were positive by qRT-PCR.

**Query 9**

Line 162 – Since this is a qRT-PCR assay, please report the quantities of viral RNA found in the supernatants of the C6/36 cells. This will help determine if the virus from the inoculated mosquito pool was infectious and replicated in the C6/36 cells.

**Answer:** We did not quantify RNA in the supernatants of the C6/36 cells. The result is based on visual fluorescence of the cell cytoplasm where viruses replicated. As we were able to isolate Dengue and Zika viruses from the mosquito pools, we can assume that these viruses were viable, and therefore infectious.

**Query 10**

Line 162 – The qRT-PCR protocol you used, as described by Patel et al, is very sensitive (10-100 genome copies). Why not determine the presence of viral RNA directly in the mosquito pools themselves?

**Answer:** We used isolation of virus in C6/36 cells to avoid false positive results, which could be a problem using only the very sensitive qRT-PCR protocol described by Patel. A further advantage of isolation in relation to the qRT-PCR protocol is that it allowed us to prove that the virus is able to replicate in C6/36 cells. In addition, the virus-isolation approach we used is cheaper.

**Query 11**

Line 206 – Technically this study doesn’t demonstrate that the mosquitoes are infected with the viruses, but that the mosquitoes contain infectious virions (if the IFA is indeed a focus forming assay). Just because there is no visible blood does not mean these mosquitoes have not blood fed recently and that some infectious virus could remain even if it isn’t infecting the mosquitoes themselves. Clarify language here and in other areas where “infected” is used

**Answer:** We thank the reviewer for pointing this out. Indeed, these viruses may have come from a recent human blood meal. However, we showed that the mosquitoes were infected as the virus was viable and could replicate in C6/36 cells.

**Query** **12**

Line 258 – Guedes et al do not demonstrate ZIKV replication in *Cx. quinquefasciatus* – a closer look at the titers of virus RNA in those mosquitoes will show RNA levels for ZIKV remaining stagnant over time and do not increase at any point measured. Clarify language

**Answer:** We respectfully disagree with the reviewer. The article by Guedes et al. [Guedes DRD et al. Zika virus replication in the mosquito *Culex quinquefasciatus* in Brazil. Emerg Microbes Infect. 2017; doi: 10.1038/emi.2017.59.] does in fact demonstrate that ZIKV replicated and was detected in the salivary glands and saliva of artificially fed *Cx. quinquefasciatus mosquitoes*.

**Query 13**

Line 262 – Your study has absolutely not demonstrated this. You have found DENV/ZIKV RNA in cell culture samples that are positive for Flaviviruses by IFA. This indicates that pools of these mosquitoes may contain virions that can infect C6/36 cells, but does not indicate that these virions are infecting the mosquitoes in the pool, or that they would be disseminated in the mosquito saliva to new hosts. This sentence greatly misrepresents your findings and should be rewritten.

**Answer:**

We agree in part with the reviewer. Our results show that the virus was infecting the mosquitoes, was viable and could replicate in C6/36. But we agree that the vector competence needs to be clarified and have modified the sentence accordingly.

**Query 14**

Line 281 – Once again, you have not found these mosquitoes to be “infected”. Clarify language

**Answer:** As before, we agree in part with the reviewer and have therefore modified the sentence as requested.

**Query 15**

Line 293 – I’m not sure one citation constitutes this mosquito being found “frequently… in natural and artificial breeding sites”. Possibly clarify this sentence or find more references

**Answer:** We agree and have modified the sentence accordingly.

**Query 16**

Line 295 – The sentence “This mosquito bites actively…” should have citations

**Answer:**

Thank you for pointing this out. We have accordingly added the reference.
